# Protective Effects of Fucoxanthin on Ultraviolet B-Induced Corneal Denervation and Inflammatory Pain in a Rat Model

**DOI:** 10.3390/md17030152

**Published:** 2019-03-05

**Authors:** Shiu-Jau Chen, Ching-Ju Lee, Tzer-Bin Lin, Hsien-Yu Peng, Hsiang-Jui Liu, Yu-Shan Chen, Kuang-Wen Tseng

**Affiliations:** 1 Department of Neurosurgery, Mackay Memorial Hospital, Taipei 10449, Taiwan; chenshiujau@gmail.com; 2 Department of Medicine, Mackay Medical College, New Taipei 25245, Taiwan; hypeng@mmc.edu.tw; 3 Internal Medicine, Taipei Hospital, Ministry of Health and Welfare, New Taipei 24213, Taiwan; lululee66@yahoo.com.tw; 4 Department of Business Administration, National Taipei University, New Taipei 24741, Taiwan; yushan@gm.ntpu.edu.tw; 5 Department of Physiology, School of Medicine, College of Medicine, Taipei Medical University, Taipei 11049, Taiwan; tblin2@tmu.edu.tw; 6 Department of Optometry, Mackay Junior College of Medicine, Nursing and Management, New Taipei 11260, Taiwan; s458@eip.mkc.edu.tw; 7 School of Life Science, National Taiwan Normal University, Taipei 10610, Taiwan

**Keywords:** fucoxanthin, ultraviolet B, denervation

## Abstract

Fucoxanthin is a carotenoid with many pharmaceutical properties that is found in brown seaweed. However, the effects of fucoxanthin on corneal innervation and intense eye pain have not been extensively examined. To clarify the protective roles and underlying mechanisms of fucoxanthin on ocular lesions, we investigated the beneficial effects and mechanisms by which fucoxanthin ameliorates ultraviolet B (UVB)-induced corneal denervation and trigeminal pain. Treatment with fucoxanthin enhanced the expression of nuclear factor erythroid 2-related factor 2 in the cornea. Inhibition of typical denervation and epithelial exfoliation in the cornea were observed in rats treated with fucoxanthin following UVB-induced nerve disorders. Moreover, the active phosphorylated form of p38 MAP kinase (pp38) and the number of glial fibrillary acidic protein (GFAP)-positive neural cells were significantly reduced. Decreased expression of neuron-selective transient receptor potential vanilloid type 1 (TRPV1) in the trigeminal ganglia neurons was also demonstrated in rats treated with fucoxanthin after UVB-induced keratitis. Symptoms of inflammatory pain, including difficulty in opening the eyes and eye wipe behaviour, were also reduced in fucoxanthin-treated groups. Pre-treatment with fucoxanthin may protect the eyes from denervation and inhibit trigeminal pain in UVB-induced photokeratitis models.

## 1. Introduction

Photokeratitis, a condition caused by exposure of the eyes to UV radiation, causes opacifications of the cornea, decreases visual acuity and induces inflammatory pain. UV-induced ocular damage has been demonstrated in previous human and animal studies [1,2]. The cornea absorbs about 90% of UVB radiation and is most sensitive to UVB impairment [3]. UVB rays irritate the superficial corneal epithelium, causing inhibition of mitosis, production of nuclear fragmentation and loosening of the epithelial layer, inducing an inflammatory response, such as stromal swelling and leukocyte infiltration [4]. UVB-induced corneal disorders could be caused by oxidative stress, as well as by the production of reactive oxygen species and inflammatory cytokines [5]. Oxidative stress may cause significant damage to cell structures and functions by increasing DNA mutations or inducing DNA damage and genome instability [6]. In addition, inflammatory cytokines have also been found to play an important role in promoting fibrosis, scarring and inflammatory pain in the cornea [7].

The transparent tissue of the cornea, which has about 2500 nerve endings per mm^2^, is one of the most densely innervated avascular tissues in mammals [8]. Its dense innervation contributes to a wide range of corneal sensations and to the maintenance of corneal epithelial integrity; touching the cornea causes an involuntary reflex that closes the eyelid. The ophthalmic branch of the trigeminal ganglion afferent nerve fibres enters the corneal stroma through the limbus and innervates the corneal epithelium [9]. Corneal nerves penetrate the corneal periphery in a radial distribution to form the sub basal nerve plexus and influence signal transduction cascades involved in epithelial function, organization and homeostasis [10,11]. It is therefore essential to conserve the cooperative interplay between the corneal innervation and epithelial microenvironments.

The trigeminal ganglia comprise neuronal cells and two types of glial cells: satellite cells and Schwann cells. The cell bodies of the primary afferent neurons are surrounded completely by several satellite glial cells that form distinct functional units. Sensitization of nociceptor fibres alters the sensitivity and pain observed during eye surface inflammation [12]. Neuron-selective transient receptor potential vanilloid type 1 (TRPV1), the capsaicin receptor primarily expressed by nociceptive sensory ganglion neurons, is directly activated and sensitized by chemical mediators released during tissue injury [13]. Previous studies have shown that the active phosphorylated form of p38 MAP kinase (pp38) is involved in cellular responses to external signals such as pain, growth factors, stress and inflammatory mediators [14,15]. Activated satellite glial cells release several inflammatory and immune mediators in response to inflammation and nerve damage and influence other sensory neurons within the ganglion [16]. The immunoreactivity against glial fibrillary acidic protein (GFAP), an intermediate filament protein, is not readily detectable in satellite glial cells in the resting state or under normal physiological conditions. However, following nerve injury, inflammation or viral infection, GFAP is detectable in the satellite glial cells of the peripheral nervous system that are activated by these pathologies. Interestingly, neuron–glia interactions have been shown to be involved in all stages of inflammation and pain associated with several diseases [17,18].

Nuclear factor erythroid 2-related factor 2 (Nrf2), a basic leucine zipper transcription factor, mediates much of the protective anti-oxidant response, regulates a coordinated transcriptional program that maintains cellular redox homeostasis and protects the cell from oxidative injury [19]. Nrf2 regulates numbers of that play important roles in the anti-oxidant enzyme response, including “direct” and “indirect” enzymes [20]. The “direct” response enzymes include catalase or superoxide dismutase and the “indirect” enzymes include heme oxygenase-1, glutathione and thioredoxin generating enzymes. Nrf2 is an important mechanistic link between managing the anti-oxidant gene expression stress response and cell survival.

Fucoxanthin is an orange pigment present in brown seaweeds, such as *Hizikia fusiformis*, *Laminaria japonica* and *Sargassum fulvellum* and is responsible for their high anti-oxidant content [21]. It is metabolized to fucoxanthinol and amarouciaxanthin in vivo [22] and has therapeutic properties, such as anti-oxidant [23], anti-cancer [24], anti-inflammatory [25], anti-obese [26], anti-diabetic [27] and anti-angiogenic activities [28] and has a protective effect on the liver, blood vessels of the brain, bones and skin. We have previously demonstrated that pre-treatment with fucoxanthin inhibited the ultraviolet B-induced expression of pro-inflammatory cytokines in the cornea [29]. However, the effects of fucoxanthin on epithelial denervation and inflammatory pain in the avascular cornea have not been extensively examined. Studies examining the reduction of corneal irregularity and inflammatory pain during corneal wound healing are of great clinical value.

## 2. Results

### 2.1. Effect of Fucoxanthin on Nrf2 Expression

**Figure 1:**
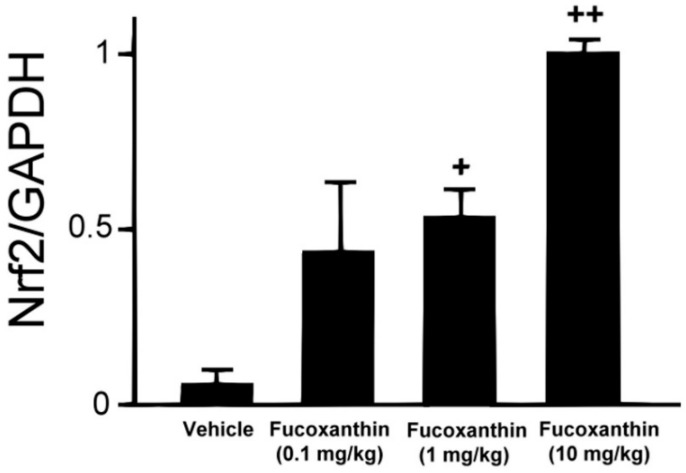
Fucoxanthin treatment enhanced the expression of Nrf2. Protein extraction was prepared and separated using SDS-PAGE, followed by western blot analysis with antibodies against Nrf2 and GAPDH. Increases in Nrf2 were observed following fucoxanthin treatment. Protein levels were measured using western blotting and band intensity was quantified using ImageJ software. The data are expressed as means ± standard deviation (SD) (*n* = 5 rats per group). + *p* < 0.05, ++ *p* < 0.01; one-way ANOVA followed by Dunnett’s multiple comparison test.

To investigate the effect of fucoxanthin on Nrf2 translocation in the cornea, experimental animals were first pre-treated with fucoxanthin ranging from 0.1 to 10 mg/kg for 6 days via oral administration. The Nrf2 protein levels were measured using western blotting analysis (Figure 1). Compared with the control group (no pre-treatment with fucoxanthin), the groups pre-treated with 0.1, 1 and 10 mg/kg fucoxanthin showed higher levels of Nrf2 protein. Moreover, Nrf2 expression increased with fucoxanthin pre-treatment.

### 2.2. Effect of Fucoxanthin on UVB-Induced Corneal Denervation

**Figure 2:**
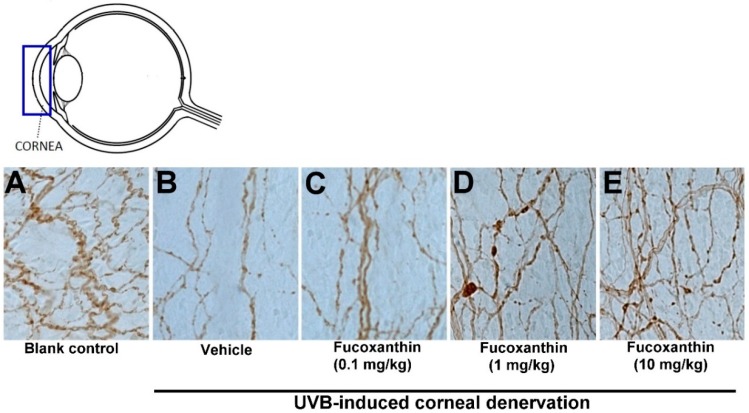
Protective effects of fucoxanthin on nerve innervation after UVB-induced denervation in corneal tissues. Nerve innervation was evaluated via immunohistochemistry using a general neural marker PGP 9.5. Nerve innervation was compared between the following groups: blank control (**A**), UVB (**B**), UVB/0.1 mg/kg fucoxanthin (**C**), UVB/1 mg/kg fucoxanthin (**D**) and UVB/10 mg/kg fucoxanthin (**E**) groups. A significant reduction in the epithelial nerve innervation of the corneal tissues is evident after UVB-induced nerve injury (**B**) compared with the blank control group (**A**). Nerve innervation was significantly protected by 10 mg/kg fucoxanthin treatment and the corneal denervation was significantly decreased.

In an attempt to define the relationship between increased Nrf2 in the ocular tissues and degenerating corneal nerve endings in the groups treated with fucoxanthin, the general neuronal marker protein gene product (PGP) 9.5 was targeted for corneal tissue staining. In the control group, abundant epithelial nerves were found in the corneas and the nerve plexus had been arranged in a crisscrossed, dense and continuous pattern of immunoreactivity toward PGP 9.5 (Figure 2A). However, fewer, discontinuous and punctate-like degenerating nerves were observed in the epithelial layer of the cornea after UVB exposure (Figure 2B). In the 10 mg/kg fucoxanthin treatment group, the experimental animals showed a significant decrease in corneal denervation and an increase in nerve density (Figure 2E) compared with those treated with 0.1 or 1 mg/kg fucoxanthin groups after UVB irradiation (Figure 2C,D).

**Figure 3:**
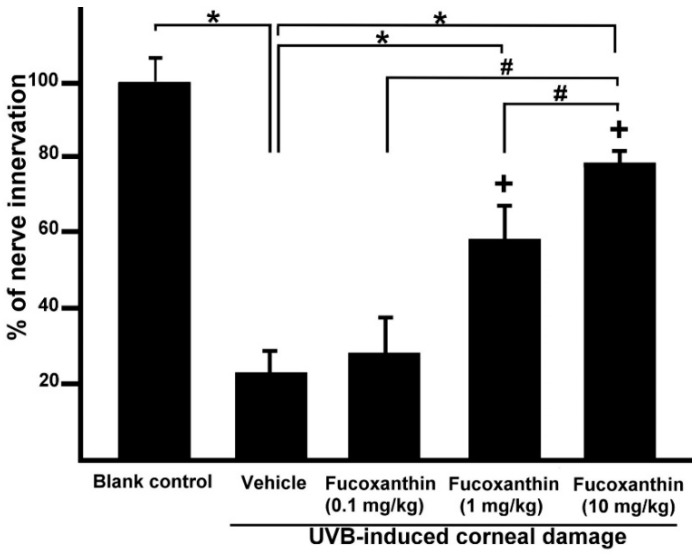
Effects of fucoxanthin on nerve innervation after UVB irradiation in the corneal tissues. Nerve innervation was compared with blank control, UVB, UVB/0.1 mg/kg fucoxanthin, UVB/1 mg/kg fucoxanthin and UVB/10 mg/kg fucoxanthin groups. A significant reduction in the nerve innervation of the corneal tissues and UVB-induced nerve injury is evident in the UVB-treated group compared with the blank control group. Nerve innervation was increased by treatment with fucoxanthin. The results are presented as means ±SD (*n* = 5 rats per group). The mean value was significantly different compared with the UVB/vehicle group (* *p* < 0.05; Student *t* test). The mean value was significantly different as UVB/0.1 and 1 mg/kg fucoxanthin groups compared with UVB/10 mg/kg fucoxanthin group (# *p* <0.05; Student *t* test). The mean value was significantly different compared with UVB-exposed group (+ *p* < 0.05; one-way ANOVA followed by Bonferroni’s multiple comparison test).

After statistical analysis of the corneal innervation, the reduction in corneal nerve density was statistically significant in the UVB group (25.3 ± 4.8%, *p* < 0.05) compared with the control. In contrast, the 10 mg/kg fucoxanthin treatment group displayed reduced denervation in the cornea (78.6 ± 5.5%, *p* < 0.05) compared with the UVB group. These findings suggest that the UVB-induced denervation in the cornea was effectively inhibited by treatment with 10 mg/kg fucoxanthin (Figure 3).

### 2.3. Effect of Fucoxanthin on UVB-Induced Corneal Epithelial Disorganization

**Figure 4:**
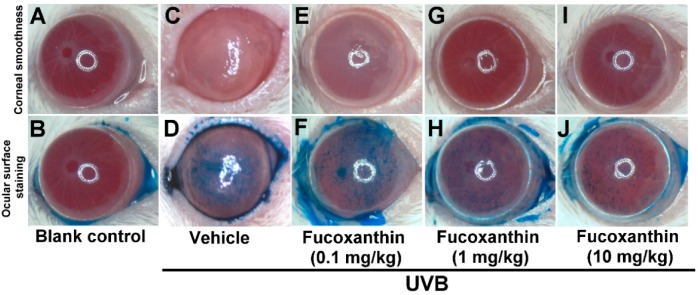
Effects of fucoxanthin on UVB-induced corneal disorders. Analyses of corneal smoothness and corneal lissamine green staining were conducted. The extent of corneal integrity and transparency was compared between the following groups: blank control (**A**,**B**), UVB (**C**,**D**), UVB/0.1 mg/kg fucoxanthin (**E**,**F**), UVB/1 mg/kg fucoxanthin (**G**,**H**) and UVB/10 mg/kg fucoxanthin. The cornea, which is normally transparent, becomes cloudy when damaged. UVB irradiation caused serious damage to the corneal tissue (**C**,**D**), including severe exfoliation, deteriorated smoothness, corneal opacity and intense staining with lissamine green, as compared with the blank controls (**A**,**B**). Corneal smoothness and surface staining analysis showed that fucoxanthin treatment preserved corneal integrity and transparency (**E**–**J**).

Because of the protective effects of fucoxanthin treatment against UVB-induced corneal denervation, the relationship between corneal denervation and pathological examinations was applied to superficial corneal inspection. Analyses of corneal smoothness and corneal lissamine green staining were conducted. The corneas of the blank control rats were transparent, regular and neat and their reflecting ocular surface was intact (Figure 4A). UVB exposure caused severe damage to the corneal epithelial surface, including distortion and deformation (Figure 4C). In contrast, corneas treated with fucoxanthin showed mild corneal epithelial disorganizations following UVB irradiation. Moreover, significant enhancement of corneal epithelial organization was observed in the groups treated with 1 and 10 mg/kg fucoxanthin (Figure 4G,I) compared with the corneal damage detected in the UVB-treated experimental animals.

The epithelial cells lining the corneal surface were stained with lissamine green to evaluate the ocular surface damage. Corneas of the blank control group remained largely unstained by lissamine green (Figure 4B), whereas signs of corneal surface disorders, including a strikingly irregular ocular surface and intense staining with lissamine green, were present after UVB radiation (Figure 4D). Treatment with 1 and 10 mg/kg fucoxanthin led to decreased lissamine green staining (Figure 4H,J). Compared with the corneal injuries detected in the UVB-treated groups, significantly fewer punctate-stained areas were observed on the ocular surfaces of the group treated with 10 mg/kg fucoxanthin (Figure 4J).

**Figure 5:**
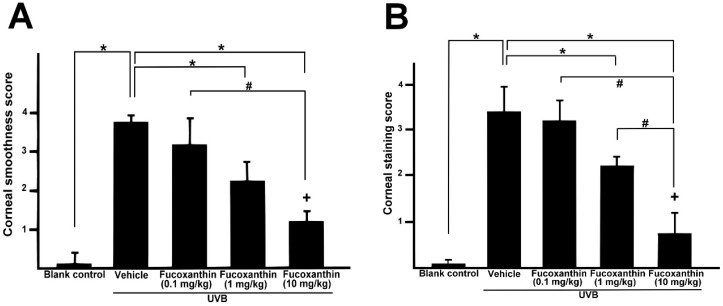
Semi-quantitative analyses of the effects of fucoxanthin on ocular surface irregularity (**A**) and lissamine green staining scores (**B**) following UVB-induced corneal damage. Data are shown as mean ± SD) (*n* = 5 rats per group). The mean value was significantly different compared with the UVB/vehicle group (* *p* < 0.05; Student *t* test). The mean value was significantly different as UVB/0.1 and 1 mg/kg fucoxanthin groups compared with UVB/10 mg/kg fucoxanthin group (# *p* <0.05; Student *t* test). The mean value was significantly different compared with UVB-exposed group (+ *p* < 0.05; one-way ANOVA followed by Bonferroni’s multiple comparison test).

Semi-quantitative analyses of corneal smoothness and lissamine green staining were scored (Figure 5) and significant differences were observed between all scores of the UVB-treated groups and those of the blank control. The corneas of the fucoxanthin-treated groups showed decreased corneal damage scores. Moreover, the analysis also revealed significantly decreased scores in the group treated with 10 mg/kg fucoxanthin as compared with the groups treated with 0.1 and 1 mg/kg fucoxanthin. Thus, the protective effects of fucoxanthin treatment against UVB-induced damage to the corneal innervation and epithelium were observed.

### 2.4. Effect of Fucoxanthin on UVB-Induced pp38, GFAP and TRPV1 Expression in the Trigeminal Ganglia

**Figure 6:**
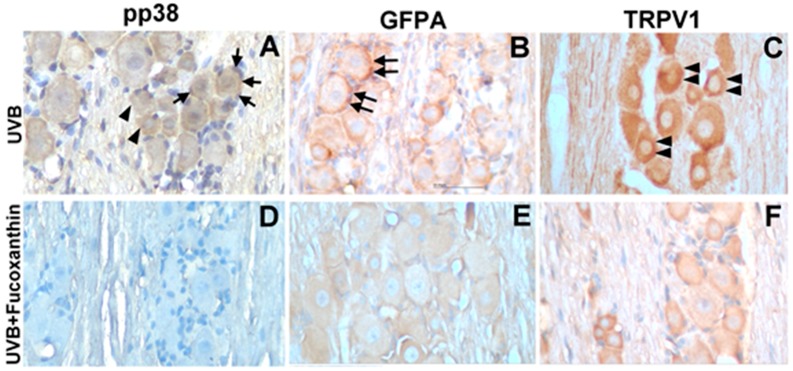
Characterization of pp38-, GFAP- and TRPV1-positive cells in the trigeminal ganglia of UVB/vehicle and UVB/10 mg/kg fucoxanthin groups. Sections were stained with haematoxylin and antibodies against pp38, GFAP and TRPV1. Expression of pp38 was increased in neuronal (arrowheads, **A**) and glial cells (arrows, **A**) in the trigeminal ganglion after UVB-induced photokeratitis. Note that the number of GFAP-immunopositive activated glial cells typically surrounding neurons (double arrows, **B**) and TRPV1-positive neuronal cells (double arrowheads, **C**) were significantly increased in the UVB/vehicle group. In contrast, the number of pp38-positive neural cells, GFAP-positive activated glial cells and TRPV1-positive neuronal cells were limited in the UVB/fucoxanthin group (**D**–**F**).

Neural activation results in increased levels of active pp38 MAP kinase, a key regulatory protein involved in signal transduction in response to inflammatory stimuli that result in changes in gene expression. To determine the relationship between UVB-induced corneal disorders and increased pp38 levels in vivo, the active levels of pp38 were monitored using immunohistochemistry. After UVB-induced photokeratitis, elevated levels of p38 were readily detected in both neurons and neighbouring satellite glial cells (Figure 6A). Moreover, the number of GFAP-immunopositive activated glial cells typically surrounding the neurons and TRPV1-positive neuronal cells were significantly increased in the trigeminal ganglion of the UVB/vehicle group (Figure 6B,C). In contrast, the number of pp38-positive neural cells, GFAP-positive activated glial cells and TRPV1-positive neuronal cells was limited in the UVB/10 mg/kg fucoxanthin group (Figure 6D–F) compared with the other UVB-treated group. These results imply that activation of satellite glial and neuronal cells was partially suppressed by fucoxanthin treatment.

### 2.5. Effect of Fucoxanthin on UVB-Induced Corneal Injury-Evoked Eye Wipe Behavior

**Figure 7:**
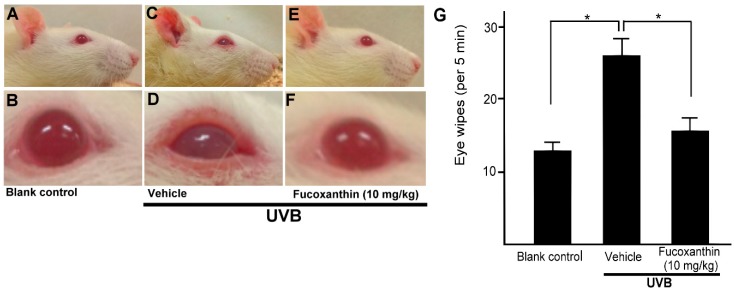
Effects of fucoxanthin on eye wipe behaviour after UVB-induced corneal damage. The cornea is composed of highly innervated tissue and pain caused by corneal damage is very intense. Photokeratitis caused inflammatory pain symptoms, including difficulty opening the eyes and eye wipe behaviour. Symptoms of inflammatory pain were compared between the following groups: blank control (**A**,**B**), UVB/vehicle (**C**,**D**) and UVB/10 mg/kg fucoxanthin treatment (**E**,**F**). Difficulty opening the eyes, the symptom of inflammatory pain, was reduced with fucoxanthin treatment (**E**,**F**). Eye wipe behaviour analysis showed a high frequency of eye wiping evoked by UVB-induced photokeratitis. In contrast, the animals showed reduced eye wiping in the UVB/10 mg/kg fucoxanthin group compared with the UVB group. The data are expressed as means ± SD (*n* = 5 rats per group). The mean value was significantly different compared with the UVB/vehicle group. (* *p* < 0.05; Student *t* test, **G**).

To assess the impact of fucoxanthin-suppressed activation of satellite glial and neuronal cells, we evaluated the symptoms of inflammatory pain and eye wipe behaviour (Figure 7). Difficulty opening the eyes, the symptom of inflammatory pain, was observed in the UVB-irradiated group (Figure 7D). Moreover, a single drop of 5 M NaCl to the ocular surface evoked consistent increases in eye wiping after UVB irradiation compared with the blank control group during a 5-min test period on day 6. The frequency of the eye wipes evoked by UVB-induced photokeratitis was high. In contrast, the animals showed reduced eye wipe frequency in the UVB/10 mg/kg fucoxanthin group compared with the UVB/vehicle group. These results indicated that UVB-induced eye wipe behaviour was suppressed in the fucoxanthin-treated group (Figure 7G).

## 3. Discussions

UVB-induced corneal disorders could be caused by oxidative stress, as well as by the production of reactive oxygen species and inflammatory cytokines [5]. The main characteristic of an antioxidant is its ability to induce enzymes that snare free radicals. In this respect, Nrf2 and its interaction with anti-oxidant response element increase the transcription that play important roles in the anti-oxidant enzyme response, such as quinone oxidoreductase 1, heme oxygenase-1 and superoxide dismutase [20]. Fucoxanthin has been shown to be able to augment cellular antioxidant defence by inducing Nrf2-driven expression in in skin cells in vivo and in vitro [30,31]. In the present study, Nrf2 expression increased with fucoxanthin treatment in corneal tissues. It is possible that Nrf2 at higher expression interact with other mechanisms to increase the activity of antioxidant in corneal tissues.

Eye signs are often associated with corneal denervation and endocrinal disorders [32,33]. Nerve innervation anomalies affect epithelium metabolism, proliferation and wound healing, compromising the health of the cornea and visual axis [32]. The corneal epithelium preserves the intraepithelial corneal nerves and the intraepithelial nerve terminals that spread out toward the apical surface [34]. The corneal nerves typically release neuronal factors that promote corneal epithelial homeostasis and effect signal transduction cascades involved in epithelial function, organization and homeostasis [10,11]. In the present study, UVB radiation caused a significant reduction in the epithelial nerve innervation of corneal tissues, resulting in evident UVB-induced nerve injury. We demonstrated that UVB irradiation impairs the density and morphology of the sensory nerves within the corneal epithelium. In addition, serious damage to the corneal tissues, including severe exfoliation, deteriorated smoothness and corneal opacity, was detected more frequently in the UVB-irradiated groups than in the non-irradiated groups. In the present study, we observed that reduced corneal nerve density was significantly correlated with the disorganization of the corneal epithelium in UVB-irradiated eyes. In contrast, treatment with fucoxanthin inhibited UVB-induced corneal denervation and preserved the integrity of the corneal surface.

UVB radiation is non-invasive, produces localized inflammation and has been applied to humans in previous studies examining cutaneous pain [35,36,37]. Ocular itching and oedema are the most frequent symptoms complained by patients not only with photokeratitis but also with keratoconjunctivitis, due to the frequent corneal involvement [38]. The inflammatory responses involved in peripheral nerve damage also include the recruitment of immune cells to the site of injury. Immune cells, including neutrophils and macrophages, are involved in the release of various kinds of pro-inflammatory mediators that contribute to pain [39,40]. The corneal epithelium, the primary protector of the optic axis and the most densely innervated tissue in the body, changes after UVB exposure and corneal inflammation is a common clinical problem that causes ocular pain. Many studies on the mechanisms of neuropathic pain have used animal models of injury to the peripheral nerve, which involves multiple complex mechanisms. TRPV1 is an essential component of the cellular signalling mechanisms through which tissue damage or infection produces thermal hyperalgesia and pain hypersensitivity is primarily expressed on small, unmyelinated sensory neurons in the trigeminal ganglia [41,42]. Recent evidence suggests that activity of the satellite glial cells connected with gap junctions and reduces the pain threshold in the trigeminal ganglion. Furthermore, inhibition of satellite glial cell activation causes a significant reduction in neuronal excitability and nocifensive reflexes [43,44]. Otherwise, the proximity of satellite glial cells and neuronal cell bodies favours interactions through paracrine signalling and contributes to the sensitization of afferent neurons. Glia and neurons form an integrated network that modulates the excitability of pain pathways. Pp38 involves both the neuronal and glial activation during inflammatory for the trigeminal ganglia activation model [15]. In the present study, aside from sensory neurons, GFAP-positive activated glial cells that typically surround neurons in the trigeminal ganglia were limited and eye wipe behaviour evoked by UVB damage was suppressed in the UVB/10 mg/kg fucoxanthin-treated group. Thus, suppressive effects of fucoxanthin on pp38, TRPV-1 and GFAP may be important in avoid inflammatory pain following photokeratitis in the trigeminal ganglia.

Previous studies have reported that neuropathic pain involves the modulation of motor activity. Neuropathic orofacial pain leads to difficulties in mandibular movements of the orofacial region, including mastication, swallowing, speaking and toothbrushing [45,46]. Following injury to the sensory branch of trigeminal nerve, not only are the number of activated satellite glial cells in the trigeminal ganglia and microglia cells in the trigeminal sensory increased but the activity of the glial cells in the trigeminal motor nuclei is also evoked [47,48,49]. It remains unclear how hyperactive glial cells interact with motor neurons. It is possible that pro-inflammatory mediators released from activated glial cells affect the excitability of motor neurons. The blink reflex is mediated by sensory fibres of the trigeminal nerve (cranial nerve V 1) and the efferent limb via the motor fibres of the facial nerve (cranial nerve VII). Anatomically, the afferent corneal V1 fibres may synapse within the main sensory nucleus and facial motor neurons in turn provide input to close the eyelids. Various ganglia and sensory and motor nuclei are involved in the feedback loops between the cornea surface and orbicularis oculi muscles. UVB-induced disorders of the cornea cause inflammatory pain and may alter the wink function. In the present study, we observed that the eyelids appeared closer together in the UVB irradiation groups than in the blank control group and that fucoxanthin treatment restored the distance between the eyelids.

Nrf2 is a transcription factor with potent antioxidant effects against cellular damage and may serve an essential role in the protection against various inflammatory diseases [50]. Otherwise, pp38 signalling pathways are associated in the regulation of the Nrf2-mediated cytoprotective effect [51]. In the present study, we demonstrated that oral pre-treatment with fucoxanthin promoted the expression of Nrf2. Exposure of the ocular surface to UVB-induced changes in corneal innervation, epithelial organization and neural activity in the trigeminal ganglia matched well with increases in the frequency of eye wipe behaviour. By contrast, significant suppression of corneal denervation and epithelial disorganization was observed in the groups treated with fucoxanthin compared with the corneal damage detected in the UVB-treated experimental animals. Activated neural cells in the trigeminal ganglia and eye wipe behaviour were also reduced and UVB-induced inflammatory pain was suppressed in fucoxanthin-treated groups. These results demonstrated that fucoxanthin treatment upregulated the expression of Nrf2, inhibited the denervation of corneal tissue and activated neural cells of the trigeminal ganglia. These findings suggested that fucoxanthin has a protective effect against UVB-induced corneal denervation and inflammatory pain and the mechanism may be associated with regulation by Nrf2 pathway in rats.

## 4. Materials and Methods

### 4.1. Experimental Animals

Fifty male SD rats (6–8 weeks old) were obtained from the Animal Department of BioLASCO Taiwan Co (Taipei, Taiwan, ROC). Experimental rats were quarantined and allowed to acclimate for 1 week before beginning experimentation. Experimental rats were housed 3–4 per cage under standard laboratory conditions with a 12-h light/dark cycle. Care and treatment of animals were conducted in accordance with standard laboratory animal protocols (IACUC-A1050023) approved by the Animal Care Committee (Mackay Medical College). Food and water were available *ad libitum*. The experimental protocols for the present study were approved by the Institutional Animal Care and Use Committee and the animals were cared for in accordance with the institutional ethical guidelines.

### 4.2. UVB-Induced Photokeratitis and Fucoxanthin Treatment

Rats were randomly divided into five groups: Group I, blank control (no UVB exposure or fucoxanthin treatment); Group II, UVB (exposure to UVB irradiation without treatment); Group III, UVB/0.1 mg/kg fucoxanthin (exposure to UVB irradiation and pre-treatment with fucoxanthin (Sigma-Aldrich, St. Louis, MO, USA) oral administration at 0.1 mg/kg body weight); Group IV, UVB/1 mg/kg fucoxanthin (exposure to UVB irradiation and pre-treatment with fucoxanthin oral administration at 1 mg/kg body weight); and Group V, UVB/10 mg/kg fucoxanthin (exposure to UVB irradiation and pre-treatment fucoxanthin oral administration at 10 mg/kg body weight). To induce corneal damage in vivo, the eyes of the rats in Groups II, III, IV and V were exposed to UVB irradiation using the procedures reported in our previous study [29]. The wavelength of the UVB source peaked at 308 nm (range, 280–315 nm). After anaesthesia with intraperitoneal injection of sodium pentobarbital (60 mg/kg bodyweight), the oculus was exposed to 550 μW/cm^2^ of UVB light (UVGL-58; UVP Inc., San Gabriel, CA) for 4 min in a darkroom. Group II (UVB) was exposed to UVB irradiation daily for a period of 5 days (Day 2 to Day 6). Groups III, IV and V (UVB/fucoxanthin at various concentrations per kg body weight) were exposed to UVB irradiation daily for a period of 5 days and treated daily with their respective concentrations of fucoxanthin (1 day before UVB exposure) for a period of 6 days (Day 1 to Day 6).

### 4.3. Tissue Fractionation and Western Blot

Rats were sacrificed by cervical dislocation after deeply anaesthesia with chloral hydrate (400 mg/kg of body weight, intraperitoneally). Corneas were rinsed once with ice-cold phosphate buffered saline (PBS) and then lysed with PBS containing 1% Triton X-100, 0.1% SDS, 0.5% sodium deoxycholate, 1 μg/mL leupeptin, 10 μg/mL aprotinin and 1 mM phenylmethylsulphonyl fluoride on ice for 15 min. After sonication for 30 s using microprobe sonicator, crude extracts were subjected to centrifugation at 4 °C. All protein concentrations were measured by a protein assay (Bio-Rad laboratories, Richmond, CA, USA). Equal amounts (50 μg) of protein extracts were separated electrophoresed on 8% SDS-polyacrylamide gel and then transblotted onto the ImmobilonTM-P membrane. After being blocked with 10% non-fat milk in Tween-20/PBS, blots were incubated with rabbit anti-Nrf2 antibody (SAB4501984; Sigma-Aldrich, St. Louis, MO, USA) and then incubated with horseradish peroxidase-conjugated secondary antibodies. The signal of specific proteins was detected using enhanced chemiluminescence kit (ECL; PerkinElmer Life Sciences, Inc. Boston, MA, USA).

### 4.4. Immunohistochemistry for Nerve Innervations in Corneal Tissues

Experimental rats were deeply anesthetized using chloral hydrate and intracardially perfused with a fixative containing 4% paraformaldehyde. A 2-mm circular corneal epithelial defect was made in each rat using a scalpel blade. For immunocytochemistry, whole-mount tissues or 50-μm cryostat sections were collected. The floating sections were rinsed in PBS, incubated in 1.5% hydrogen peroxide for 45 min to eliminate endogenous peroxidase activity and finally blocked for 1 h in PBS containing 5% goat serum and 0.5% Triton X-100 (Sigma-Aldrich, St. Louis, MO, USA). Nerve innervation was evaluated via immunohistochemistry using antibodies against general neural marker protein PGP 9.5 (AB1761, Chemicon, Temecula, CA, USA). The reaction products of nerve profiles in the cornea were evaluated using a Vector ABC kit and the 3,3-diaminobenzadine (DAB) reaction (Vector Labs, Burlingame, CA, USA). PGP 9.5-immnopositive nerve fibres in the epithelium of each cornea were counted at a magnification of 400× with a light microscope. Nerves were traced and the total length calculated with commercial software (Adobe Illustrator; Adobe Systems, San Jose, CA, USA) using an object-length function was reported in a previous protocol [52], with slight modifications. The density of the nerves was then charted as a percentage of the control.

### 4.5. Determination of Corneal Injuries

Rat corneal damage was appraised as in our previous study [29]. A circular illumination source was attached to a stereoscopic microscope (Stemi DV4 Stereo Microscope, Carl Zeiss, Oberkochen, Germany), which was then used to evaluate the corneas. Ocular surface irregularity was graded on a scale of 0–4 as follows: grade 0, absent; grade 1, mild; grade 2, moderate; grade 3, severe with a twisted circle shape; and grade 4, severe with an undistinguished shape. After corneal smoothness was scored, the ocular surface was stained with 1% lissamine green (Sigma-Aldrich, St. Louis, MO, USA) and washed three times with PBS. Images of the corneal surface after lissamine green staining were taken and scored according to the grading system based on the proportion of staining noted in the damaged cornea. The severity of corneal damage was graded on a scale from 0 to 4. Briefly, the total area without dot staining was designated as grade 0; grade 1, less than 25% of the corneal surface was stained with dotted punctuate staining; grade 2, 25–50% of the corneal surface was stained with diffuse punctuate staining; grade 3, 50–75% of the corneal surface was stained with punctuate staining and apparent corneal defects; and grade 4, more than 75% of the corneal surface was stained with profuse punctuate staining and more serious corneal defects. Corneal scoring was performed by three observers who had no prior knowledge of photokeratitis.

### 4.6. Immunohistochemistry of pp38, TRPV1 and GFAP in Trigeminal Ganglia

Whole trigeminal ganglia were fixed in 4% paraformaldehyde for 45 min, embedded in media (Miles, Elkhart, IN, USA), maintained at the optimal cutting temperature and frozen in isopentane supercooled with liquid nitrogen. Using a cryostat (Leica Microsystems, Heidelberg, Germany), 8-μm cryosections were cut and collected on silanized slides (DAKO, Tokyo, Japan). For immunohistochemical staining of the trigeminal ganglion, serial sections were reacted with rabbit anti-activated pp38 MAP kinase anti-serum (ab47363; Abcam, Cambridge, MA, USA), rabbit anti-GFAP (ab7260; Abcam, Cambridge, MA, USA) or rabbit anti-TRPV1 anti-serum (ab6166; Abcam, Cambridge, MA, USA) after dilution in PBS containing 4% normal goat serum and 0.3% Triton X-100 (Sigma-Aldrich, St. Louis, MO, USA). Sections were then reacted with biotinylated goat anti-rabbit IgG (Vector Laboratories, Burlingame, CA, USA), avidin and biotin-peroxidase complex (Vector Laboratories) and visualized with 0.1% DAB.

### 4.7. Eye Wipe Behavior Test

The eye wipe test was performed using hypertonic saline as the inducing stimulus following a previous protocol [53], with modifications. This is a sensitive test for acute ocular irritation-related behaviour in conscious rats that is non-invasive and non-inflammatory. A single drop of NaCl (5 M, 20 μL) was placed on the ocular surface with a micropipette and the number of eye wipes with the ipsilateral forelimb that occurred in 30 s was counted on day 6. Means and standard deviations were calculated for all parameters determined in this study. Comparison analyses between any two groups were performed using Student’s *t*-test. A *p*-value < 0.05 was considered statistically significant.

### 4.8. Statistical Analysis

Statistics were analysed using the SPSS program (SPSS, Inc., Chicago, IL, USA). Means and standard deviations were calculated for all parameters determined in this study. In all samples, Kolmogorov-Smirnov test was used to verify the normality of the data. The non-parametric values were analysed by Mann–Whitney test. The parametric values groups were analysed by one-way analysis of variance (ANOVA) followed by Dunnett’s or Bonferroni’s multiple comparison test. Statistically significant differences between groups were defined as *p* < 0.05.
